# Pre‐Screening in Clinical Trials: Incentives, Behaviours, Consequences

**DOI:** 10.1111/jep.70231

**Published:** 2025-07-31

**Authors:** Richard C. Armitage

**Affiliations:** ^1^ Academic Unit of Population and Lifespan Sciences, School of Medicine University of Nottingham Nottingham Nottinghamshire UK

## 1 Randomised Controlled Trials (RCTs)

RCTs are prospective studies with the ability to isolate the effect of an intervention by controlling for confounding factors through randomisation and blinding, which eliminates much of the bias inherent to other study designs [1, 2]. This methodological rigour ensures high internal validity and positions RCTs as the reference standard for studying causal relationships between interventions and outcomes.

One major limitation of many contemporary RCTs is their relatively low external validity. This is largely due to systematic exclusion of substantial proportions of the patient population through highly selective and narrowly defined trial inclusion and exclusion criteria. This exclusion takes place at the screening stage and often results in older adults and patients with multiple comorbidities, polypharmacy, cognitive impairment, or a history of drug reactions being routinely omitted from trials [3]. While such methodologies are designed to promote patient safety and minimise variability, these restrictions generate trial results with limited generalisability to the wider patient population [4], including to those who stand to receive the greatest benefit from the intervention [5].

## 2 Pre‐Screening in Clinical Trials

Participant screening is the formal process in which potential trial participants are considered for recruitment by evaluation against the protocol's inclusion and exclusion criteria. Pre‐screening, however, is the informal step that occurs before formal screening that determines which potential participants are invited to formal screening. It has been noted that pre‐screening activities are broadly unstandardised across trial sites and go largely undocumented and unreported by investigators in the scientific literature [6]. The consequence of these behaviours is the systematic exclusion of large proportions of the patient population, many of whom would satisfy the protocol's inclusion and exclusion criteria and be eligible for trial participation if they had progressed to formal screening. This ‘hidden layer’ further confines study populations and goes largely unrecognised in academic discourse [7].

## 3 Incentives

Examining the incentives that surround the pre‐screening stage of clinical trials can help to explain investigator behaviours and elucidate their consequences, and facilitate the offering of recommendations for improvement.

Several factors influence investigators during the pre‐screening stage: study teams face pressure to recruit a pre‐committed minimum number of participants by specified deadlines as agreed with sponsors; competition exists between study sites, whether in the same country or internationally, to meet sponsor expectations for participant recruitment; investigators are motivated to recruit participants quickly to enhance their site's reputation for effectiveness, making them more attractive to sponsors for future trial selections; pre‐screening activities are typically not compensated by sponsors; and formal screening requires substantial staff time and consumable resources that often go unreimbursed by sponsors. These factors create substantial opportunity costs when screening less favourable potential participants. Additionally, sponsors frequently penalise investigators for formal screening failures, creating further pressure to be selective during the pre‐screening stage.

## 4 Behaviours

This combination of incentives drives investigator behaviours in predictable ways such that only potential participants that are highly likely to enrol in and complete a trial are invited to formal screening. These behaviours manifest in two major ways.

First, because potential participant pools often consist of tens of thousands of individuals, and as sponsor expectations require rapid recruitment of participants, pre‐screening decisions are usually based on the relatively little information provided by potential participants' electronic health records. These might be further limited due to information‐sharing restrictions between third parties, such as between the potential participant's registered GP practice and the trial site. Furthermore, the requirement for speed often leads to pre‐screening decisions without direct communication with potential participants. Accordingly, the quality of these decisions is often low.

Second, pre‐screening decisions are often made not only according to the protocol's inclusion and exclusion criteria, but also based on various additional factors. These include anticipated barriers to participation and predicted non‐adherence such as cognitive challenges, language barriers, unstable living conditions, perceived lack of motivation, [7] previous non‐attendance at medical appointments, travel time to the trial site, historical evidence of mental health problems, having young children, carer status, the presence of somatoform disorders, and historical safeguarding concerns. In addition, further factors also inform pre‐screening decisions, including the following: participants who completed previous trials might be invited to screening for new ones due to their proven ability to adhere to protocols; trials might be selectively advertised to specific groups regarded as being enthusiastic about the value of trials and willing to participate in them, such as healthcare students; since participation in multiple trials simultaneously is prohibited, participants might be ‘saved’ for other forthcoming trials despite the current trial being more suitable for the participant; and, advertising of trials might be timed to prioritise transient populations, such as university students during term time.

Accordingly, pre‐screening decisions are often relatively uninformed and lacking in nuance. Furthermore, the rationale that underlies these decisions largely go undocumented such that screening decisions often operate as a ‘black box’ that is concealed from the scientific community [7].

## 5 Consequences

These investigator behaviours in the pre‐screening stage generate two main negative consequences. First, many groups beyond those determined by the protocol's inclusion and exclusion criteria are underrepresented in clinical trial participation, which further weakens the trial's generalisability to the broader patient population. This also undermines the ethical principle of justice, which requires doctors to ensure that the benefits and costs of actions are fairly distributed between patients [8]. Second, many individuals who would be eligible to participate in clinical trials, are eager to do so, and stand to benefit from the intervention under investigation are inappropriately excluded at the pre‐screening stage, which undermines the ethical principles of respect for autonomy and beneficence [8].

## 6 Recommendations for Practice

It is important to recognise that these consequences of investigator pre‐screening behaviours are not due to investigators' methodological or moral failings, but are rational actions that result from perverse incentives. Accordingly, by reimagining these incentives, the downstream behaviours could be altered and their negative consequences mitigated.

One recommendation that would most heavily impact the formal screening stage is for investigators and sponsors to design trials with eligibility criteria that are less tightly selective such that the trial population bears a closer resemblance to the general population [7]. Recommendations with specific regard to the pre‐screening phase include the following:

First, pre‐screening decision‐making should be standardised across all sites conducting a specific trial; second, all pre‐screening decisions, and the reasoning that underlies them, should be transparently documented by investigators; third, sponsors should remunerate trial sites for their pre‐screening activities and expended consumable resources, regardless of enrolment outcome; fourth, sponsors should eliminate screening failure penalties; fifth, when reported, the results of clinical trials should include the number of potential participants who were pre‐screened and the associated reasons for exclusion [6], and academic journals should require this information to be included in the manuscripts reporting clinical trials that are submitted for publication. To improve and standardise these reporting standards, the CONSORT 2025 statement—a widely accepted checklist of the minimum set of items to be included in a clinical trial report and a flow diagram for documenting the flow of participants through a trial [9]—should be adapted to require this pre‐screening information. ‘Pre‐screening eligibility criteria’ should be added to the ‘Eligibility criteria’ section (number 12) in the checklist, and ‘n excluded during pre‐screening’ should be added to the Enrolment section of the flow diagram at the top of the ‘n excluded’ box.

## Conflicts of Interest

The author declares no conflicts of interest.

## Data Availability

Data sharing is not applicable to this article as no new data were created or analysed in this study.
